# The influence of limb role, direction of movement and limb dominance on movement strategies during block jump-landings in volleyball

**DOI:** 10.1038/s41598-021-03106-0

**Published:** 2021-12-08

**Authors:** Elia Mercado-Palomino, Francisco Aragón-Royón, Jim Richards, José M. Benítez, Aurelio Ureña Espa

**Affiliations:** 1 Department of Physical and Sport Education, Faculty of Sport, Human Lab - Sport and Health University Research Institute (iMUDS), University of Granada, Carretera de Alfacar, s/n, 18011 Granada, Spain; 2 Department of Computer Science and Artificial Intelligence, DICITS, DASCI, IMUDS, University of Granada, Granada, Spain; 3 Allied Health Research Unit, University of Central Lancashire, Preston, UK

**Keywords:** Risk factors, Musculoskeletal system

## Abstract

The identification of movement strategies in situations that are as ecologically valid as possible is essential for the understanding of lower limb interactions. This study considered the kinetic and kinematic data for the hip, knee and ankle joints from 376 block jump-landings when moving in the dominant and non-dominant directions from fourteen senior national female volleyball players. Two Machine Learning methods were used to generate the models from the dataset, Random Forest and Artificial Neural Networks. In addition, decision trees were used to detect which variables were relevant to discern the limb movement strategies and to provide a meaningful prediction. The results showed statistically significant differences when comparing the movement strategies between limb role (accuracy > 88.0% and > 89.3%, respectively), and when moving in the different directions but performing the same role (accuracy > 92.3% and > 91.2%, respectively). This highlights the importance of considering limb dominance, limb role and direction of movement during block jump-landings in the identification of which biomechanical variables are the most influential in the movement strategies. Moreover, Machine Learning allows the exploration of how the joints of both limbs interact during sporting tasks, which could provide a greater understanding and identification of risky movements and preventative strategies. All these detailed and valuable descriptions could provide relevant information about how to improve the performance of the players and how to plan trainings in order to avoid an overload that could lead to risk of injury. This highlights that, there is a necessity to consider the learning models, in which the spike approach unilaterally is taught before the block approach (bilaterally). Therefore, we support the idea of teaching bilateral approach before learning the spike, in order to improve coordination and to avoid asymmetries between limbs.

## Introduction

In volleyball, when a player is trying to get the greatest spike performance they use a three-step sequence which is determined by the dominant hand which performs the hit^1^. Hence, players are used to landing with their non-dominant limb when a spike is performed. Similarly, the limb role depends on which limb starts the three-step approach, leading the sequence. For example, for a left-handed player, the usual three-step approach during a spike should be right–left–right, which should be the same sequence as a block jump-landing when moving to the left side (moving to zone II), and therefore moving to the dominant direction. In this case, his non-dominant limb (the right one) is also the lead limb, because it is the first one to initiate the three-step approach, and therefore the left limb is also the trail limb. Thus, the direction of the block jump-landing will vary within the game situation, resulting in a change to their normal three-step sequence when moving to the non-dominant direction, which in turn will affect the jump-landing movement strategy. This can produce different limb movement strategies during jump-landing, and subsequently highlights possible asymmetries in strength and balance^2^.

Muscle imbalance has been shown to be useful in the identification of athletes at risk of lower limb injuries. These may be associated with strength differences^3^, side to side differences due to incomplete or improper recovery from an injury^4,5^, or repetitive limb use^2^. Muscle loading patterns experienced around the knee may alter the balance of muscle strength under high velocity conditions^2^. However, little is known regarding the influence that leg preference or playing position may have on lower-extremity muscle strength and asymmetry^3^. Therefore, there is a necessity to study the differences in movement strategies considering both the dominant and non-dominant directions and limb role during training and match situations.

Volleyball-specific tasks such as jumping, landing, blocking and spiking the ball need to be combined with fast directional movements, which produces a great demand on the musculoskeletal system^6^. As a consequence, volleyball players are at risk of musculoskeletal injuries^6^. It has been reported that the hip, knee and ankle are the most commonly injured joints in volleyball^7^. Injuries appear to occur most often just after the initial contact with the ground or during passive loading when the impact peak occurs^8^. The effectiveness of block jump-landings can be related to anticipation, movement speed, decision-making and jumping ability^9^. However, when a volleyball player is performing a block jump-landing efficiently, they move into tibial internal rotation which can lead to increased knee abduction and greater anterior cruciate ligament (ACL) loading^10^. Moreover, when the foot is fixed on the ground, lateral trunk bending can result in an external hip abduction moment, which needs to be balanced by an internal hip adduction moment from the hip adductor muscles. This may cause the knee to move medially and increase external knee abduction moments during landing^11^. Previous studies indicate that it is highly probable that lower limb injuries are more likely to involve multi-planar rather than single-planar mechanisms^12^. Notwithstanding, it has also been suggested that angular velocities in all three planes may be a better measurement of lower limb control^13^, which have also been related to force generation and muscle activity^14^.

Some studies have analysed the different variables associated with lower limb injury risk by considering the biomechanics of jump-landings in volleyball^15–18^, however the protocols which have been used still do not accurately represent real game situations. The majority of previous work has not considered both limbs, velocity of movements, jumping distance, three-step sequence, movements to the dominant and non-dominant directions, the limb role, or the movement of the joints of the lower limbs in 6 degrees of freedom. To the authors’ knowledge, no investigation exists which considers all these points during block jump-landings. We believe that it is necessary to introduce a natural volleyball block jump-landing technique including arm swing^19^, a three-step sequence technique^9^ and movements to both the dominant and non-dominant directions^17^, which should be performed as fast as possible to provide a closer representation of a game situation. Lobietti et al.^20^ highlighted the importance of standardizing conditions including; directions, distance, and height of the jumps so that players land in a manner closer to that seen during a competition.

The consideration of as many relevant risk factors as possible is necessary to understand the movements during the multifactorial nature of sports injuries^21^. However, the analysis of all these variables requires the utilization of complex methods of data analysis. Machine Learning is a subfield within Artificial Intelligence (AI), this is based on methods which are able to automatically learn complex patterns inherent in a dataset and apply them to new data to predict future behaviour. As a result, these can be applied to the classification of tasks by assigning a class or a label to new data based on what has been previously learned. The number of papers which have used Machine Learning to gain an improved perspective of a larger number of variables and how they are related is increasing. A recent systematic review suggested that the application of AI methods in team sports has the potential to grow further, with the continued development and application of “on field” evaluations within sports to establish the predictive performances of different techniques^22^. Moreover, Cust et al.^23^ demonstrated the capacity of such Machine and Deep Learning methods to improve the understanding of sport movements and skill recognition, and how this can be applied to performance analysis to automate sport-specific movement recognition^23^.

This current study explores the use of two Machine Learning methods: Artificial Neural Networks (ANN)^24^ and Random Forest (RF)^25^, with the aim to classify conditions for the directions of movement and limb role using kinematic and kinetic data, and decision trees to determine which variables were relevant to discern any differences in limb movement strategies. This aims to address the limitations of previous studies, by creating a protocol which considers all the relevant variables in an ecological situation to allow a better understanding of any differences. Therefore, the objectives of this study were to determine if significant differences exist between movements in the dominant and non-dominant directions, between the lead and trail limbs and between the dominant and non-dominant limbs during block jump-landings. And specifically, to determine which significant differences were between the lead and trail limb when moving to the dominant direction (question 1) and to the non-dominant direction (question 2), and between the dominant and non-dominant limb when both are performing the lead role (question 3) and when both are performing the trail role (question 4). So, an additional goal is to determine if the use of Machine Learning offers an analysis method capable of identifying different motor patterns during sporting tasks.

## Method

### Study design

This study is a within-subjects design where the independent variables were (Table 1):

**Table 1:** Limbs related variables according to their limb dominance.

	Directions of movement	Role	Limb	From zone III to
Right-handed player	Dominant	Lead	Non-Dominant	Zone IV
		Trail	Dominant	
	Non-Dominant	Lead	Dominant	Zone II
		Trail	Non-Dominant	
Left-handed player	Dominant	Lead	Non-Dominant	Zone II
		Trail	Dominant	
	Non-Dominant	Lead	Dominant	Zone IV
		Trail	Non-Dominant	

(1) Limb dominance: The dominant limb was determined as the preferred leg to kick a ball^26^, which was the same as the preferred arm, with thirteen right-handed and one left-handed players; (2) Directions of movement: movement to the dominant direction was considered as the direction in which the participant performed their normal three-step sequence used when performing a volleyball spike; and (3) Limb role: the lead limb was defined as the ipsilateral limb and the trail limb defined as the contralateral limb during the jump-landing. For instance, for a right-handed player, when moving to the dominant direction, their dominant limb (right limb) corresponds with the trail limb, but contrarily when moving to the non-dominant direction, their dominant limb corresponds with the lead limb.

The dataset is composed of data representing each jump which is labelled for each limb. For example, for question 1, the jump may be labelled as either “dominant” or “non-dominant” direction. Each of the four problems is actually a binary classification problem, where the goal of the classification model is to compute an answer for the data representing a jump, so that it predicts the correct label. So, the model outputs can be easily described through a confusion matrix of size 2 × 2, with true positive meaning that the model predicted correctly the jump label.

### Subjects

Fourteen female senior national volleyball players; aged 20.43 ± 2.17 years, height 171.24 ± 3.3 cm, mass 65.65 ± 6.34 kg and who played in a national league participated in the study. Any participants who had a history of hip, knee or ankle surgery within the previous 6 months were excluded. This study was approved by the University of Granada ethics committee in accordance with the Declaration of Helsinki. Prior to testing, the aims of the study and the experimental procedures were explained to the participants who then signed an informed consent form. The dataset intends to represent reality so the rate of left-handed players is close to the rate of left-handed prevalence in the world population (roughly 10%). Anyway, this is not a limitation of the study since it is focused on the dominant limb not on whether the player is right- or left-handed.

### Variables

The variables considered in the classification machine learning methods included; hip, knee and ankle angles (deg), angular velocities (deg/s) and joint moments (Nm/kg) in the sagittal, coronal and transverse planes, and joint power absorption (J/kg) in the sagittal plane. In addition, the vertical ground reaction force (N) and loading rate (N/s) for each limb were also included. Most of the biomechanical variables that have been previously reported in literature as risk factors in lower limb injuries in all planes^10–18^ have been chosen.

The measurements of 32 variables from 376 block jump landings from both limbs were analysed between initial contact (the first occurrence of a ground reaction force > 20 N on each platform) and the maximum knee flexion moment^18^. The input data for the Machine Learning methods correspond with the first Vertical Ground Reaction Force peak (F1), just after the initial contact for each trial and each limb, due to this peak was considered to be related to injury’s risk^8^. All the data analysis were performed using the R statistical software (R version 3.4.4). The experimental data was represented as a matrix of 752 rows by 32 columns. Each row was labelled according to: (1) limb dominance, (2) direction of movement, and (3) limb role. In the first scenario, row data corresponding for each volleyball player was kept grouped. In the second, this information was not considered.

### Experimental setup

Ground reaction force data were collected at a sampling rate of 250 Hz using two force platforms (9260AA Kistler Instruments, Hampshire, UK) embedded in the floor. Synchronously, an eight camera Oqus motion capture system (Qualisys, Sweden) was used to collect kinematic data at a sampling frequency of 250 Hz. Twenty one retro-reflective markers were placed on each subject prior to data collection^27^. Moreover, FitLight Trainer lights were used (Fitlight Sports Corp., Canada) in order to simulate an attack and to determine if the block was high enough to be effective.

### Protocol

**Figure 1:**
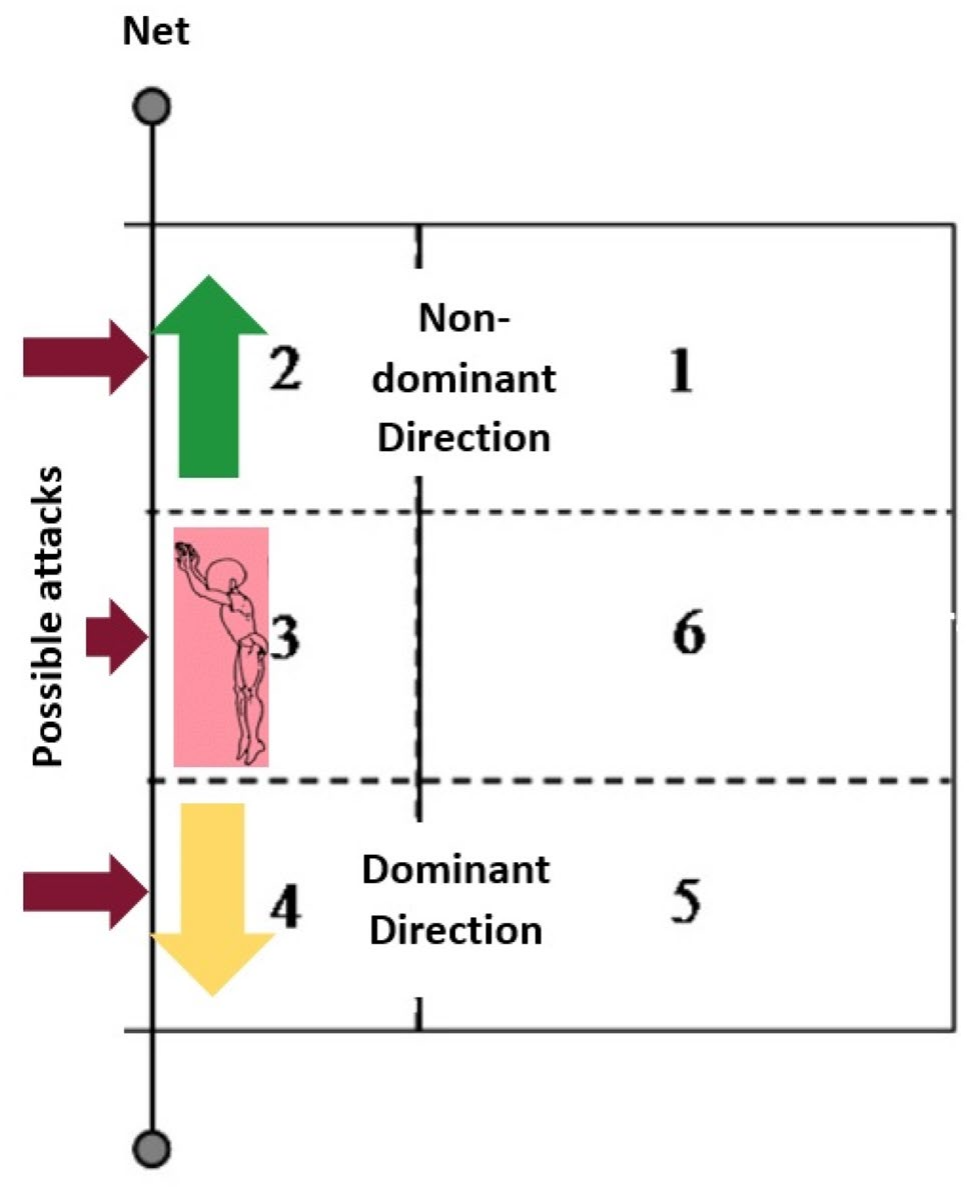
Example of a right-handed player performing a block jump-landing when moving in the non-dominant direction (moving to zone II), and when moving in the dominant direction (moving to zone IV).

The experimental setting was based on a real game situation with the upper edge of the net set at 2.24 m. The height of the jump was normalised holding a FitLight in the space located 0.20 m above the edge of the net and on the opponent’s side of the court. These lights were used as a target to create visual reaction information to the player, such as showing the blocking direction whilst checking that the block has been made at the correct height. A block was considered successful when the jump-landing was as fast as possible by an evaluator, the player arrived at the light to turn it off and both limbs landed on the force platforms which were embedded in the floor. Despite participants knowing the location of the force platforms, it was explained to them that they were not to target these. All trials which did not accomplish these characteristics were discarded. To measure directions, the players started the block approach 3 m away from the force platforms from the left and the right sides (Fig. 1), simulating when moving to zone IV and to zone II in a normal game. Hence, the jump landing tasks were as realistic as possible to increase the ecological validity of the protocol, due to it was considered velocity, approach distance and the side direction.

The trials were performed in a single session during the course of 1 day. Before data collection, all subjects performed a 20 min warm-up consisting of stretching the lower and upper extremities. Five training attempts followed the warm-up. At the start of each trial, the subject performed a block jump-landing, from the left or right side, the direction of which was randomized. The participants were informed that they had to go at full speed and block the simulated attack. A rest of 5 min was allowed after each sequence and the protocol was repeated from the opposite direction, with at least twelve successful attempts performed under each condition. The Borg scale 6–20 was recorded after each sequence to control for fatigue and was maintained under the threshold of fifteen points.

### Analysis, model training and testing

The marker data were processed using Qualisys Track Manager (QTM, Qualisys Inc., Gothenburg, Sweden) and exported into c3d format. Visual3D (C-Motion, Inc., Rockville, MD, USA) was used to calculate the three-dimensional ankle, knee and hip kinetics and kinematics.

It was used 11 players for training and 3 players for testing. The training data were used to fit and tune the Machine Learning models, while the test data were used to evaluate the performance of the fitted models. All the data features were numeric and there were no missing values. All data were normalized (centred and scaled) using the interval [0–1] for each model, where the minimum value was mapped to 0 and the maximum value to 1. Different measures have been used to assess the performance of the ML models: accuracy, sensitivity specificity, precision, recall and F1-score. The accuracy (ACC) was used as the leading measure of performance of the models using the test data, where 1 would correspond with 100% efficiency. ACC is represented as the proportion of correctly classified instances among the total number of test instances. These are commonly used measures with standardized definitions, which can be found in any introduction manual to Machine Learning or introductory papers, such as Taborri et al. (2021)^28^.

Two Machine Learning methods—selected from the current state-of-the-art—were used to generate the models from the dataset, Artificial Neural Networks (ANN) and Random Forest (RF). These were used to classify differences between conditions for limb dominance and limb roles from the kinematic and kinetic data. Both techniques implement the supervised learning paradigm. The ANN was implemented using the mlp function of the RSNNS R package. A multilayer perceptron (fully connected feed-forward network) with 3 layers (input, hidden and output) and sigmoid activation function was used. In addition, different sizes of the hidden layer (3, 5 and 7) and the learning rate parameter (0.1, 0.15, and 0.2) were used during the training. The RF were implemented using the RRF function of the RRF R package. The RF algorithm was used without regularization and with a variable number of trees (100, 200, 300, 400 and 500).

The performance of the Machine Learning methods depends on several hyperparameters, specific for each method (specify above). To select the best combination of these parameters a grid search was carried out based on a tenfold cross-validation on the training data and the higher average ACC values were selected. A model with these combinations of hyperparameters was then used to fit the training dataset. These were then used to perform the prediction of the classification on the test.

ANN and RF strive for the best accuracy, but lack in interpretability, therefore we also used decision trees which construct easier to understand models. In particular, they perform an implicit feature selection reducing the complexity of the model. The decision trees were adjusted using some R package (RPART, party, C50 and tree). The decision trees were painted based on the best model of the package with a better accuracy.

### Ethics approval and consent to participate

This study was approved by the University of Granada ethics committee in accordance with the Declaration of Helsinki. Prior to testing, the aims of the study and the experimental procedures were explained to the participants who then signed an informed consent form.

### Consent for publication

We hereby declare, that there was no conflict of interest associated with our submission of the manuscript.

## Results

**Figure 2:**
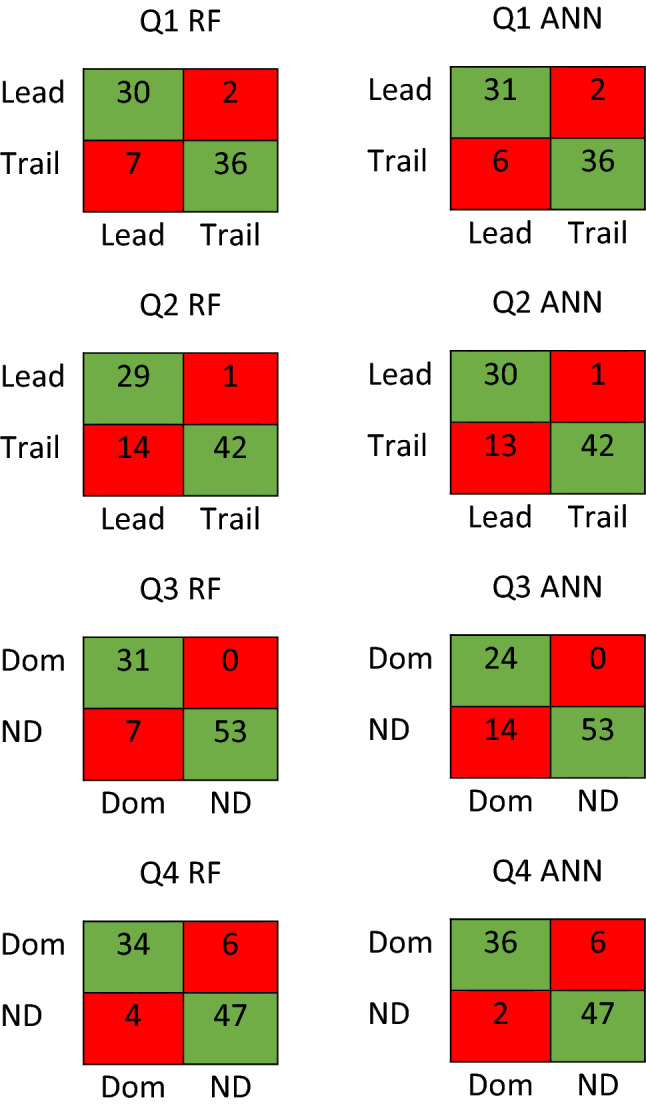
Confusion matrices calculated for all the test results. RF: Random Forest; ANN: Artificial Neural Network; Q1: Question 1; Q2: Question 2; Q3: Question 3; Q4: Question 4; Lead: Lead limb; Trail: Trail limb; Dom: Dominant limb; ND: Non-dominant limb.

Table 2 shows test results of accuracy, sensitivity, specificity, precision, recall and F1-score for each model when trained on data from variables for each question. Moreover, Fig. 2 shows the confusion matrices calculated for all the test results. When comparing between limbs in the jump-landings different movement strategies were seen between the lead and the trail limb with a predictive accuracy > 89.3%. In addition, when comparing between limbs when moving in the different directions performing the same role differences in movement strategy were seen with a predictive accuracy > 92.3%.

**Table 2:** Test results of accuracy, sensitivity, specificity, precision, recall and F1-score for each model when trained on data from variables.

	Accuracy	Sensitivity	Specificity	Precision	Recall	F1-score
**Q1**						
RF	0.8800	0.8108	0.9474	0.9375	0.8108	0.8696
ANN	0.8933	0.8378	0.9474	0.9394	0.8378	0.8857
**Q2**						
RF	0.8256	0.6744	1.0000	1.0000	0.6744	0.7945
ANN	0.8372	0.6977	0.9767	0.9677	0.6977	0.8108
**Q3**						
RF	0.9230	0.8158	1.0000	1.0000	0.8158	0.8986
ANN	0.8462	0.6316	1.0000	1.0000	0.6316	0.7742
**Q4**						
RF	0.8901	0.8947	0.8868	0.8500	0.8947	0.8718
ANN	0.9121	0.9474	0.8868	0.8571	0.9474	0.9000

*RF* random forest, *ANN* artificial neural network, *Q1* Question 1, *Q2* Question 2, *Q3* Question 3, *Q4* Question 4.

**Figure 3:**
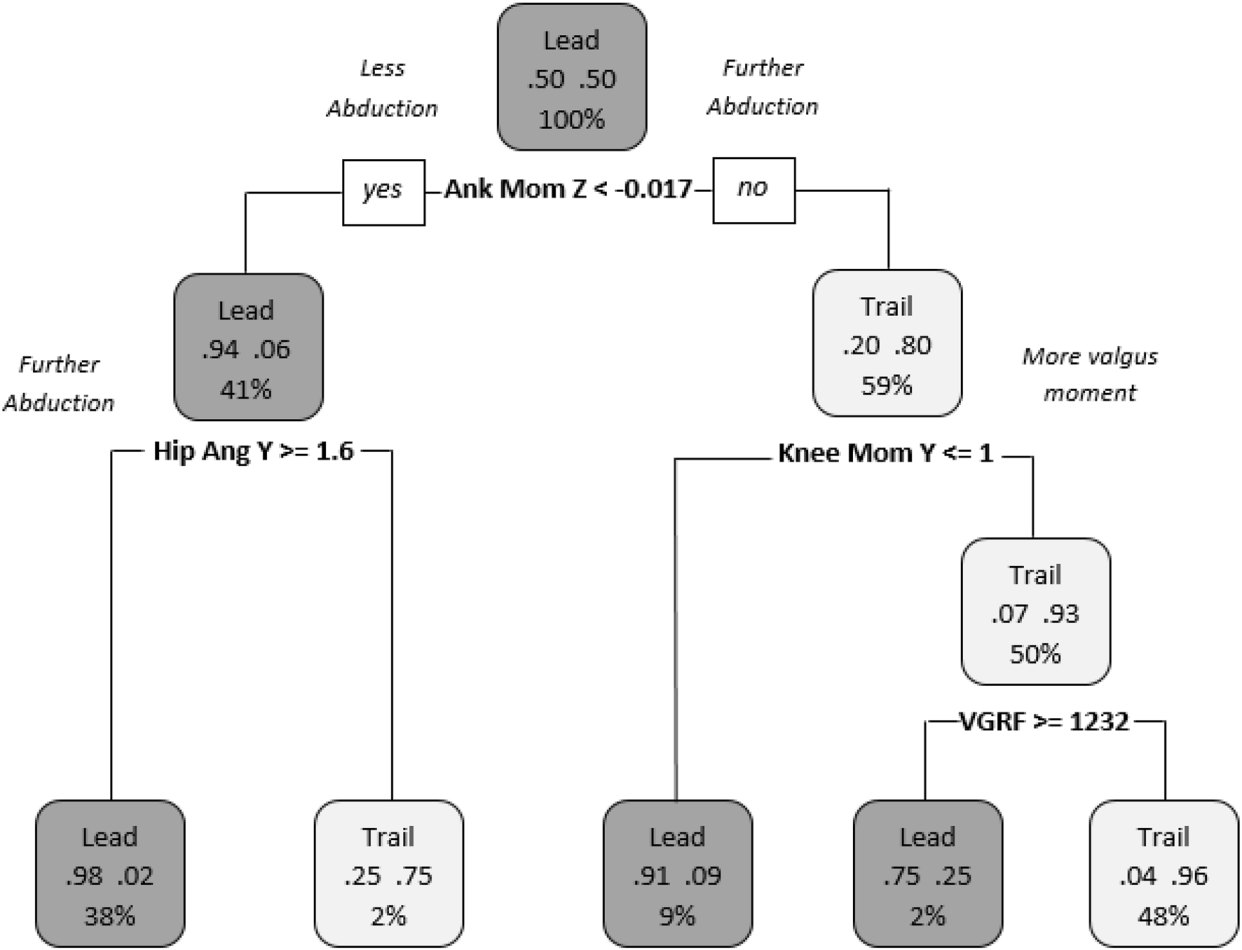
Differences between the lead and trail limbs in jump-landings when moving in the dominant direction. “Lead”: lead limb; “Trail”: trail limb; “Ank Mom Z”: Ankle moment in the transverse plane; “Hip Ang Y”: Hip angle in the coronal plane; “Knee Mom Y”: Knee moment in the coronal plane and “VGRF”: Vertical Ground Reaction Force.

Question 1 considered if significant differences exist between the lead and trail limbs in jump-landings when moving in the dominant direction. Difference in strategy between limbs were identified with a predictive accuracy of 89.33% with both Machine Learning methods. Figure 3, shows the decision tree used to explore the lead limb strategy which tends towards a lower abduction ankle moment in the transverse plane and a higher abduction hip angle in the coronal plane in 38% of trials. In addition, in 48% of trials the trail limb strategy tended towards a higher abduction ankle moment in the transverse plane, a higher knee valgus moment in the coronal plane and a lower peak vertical ground reaction force than the lead limb.

**Figure 4:**
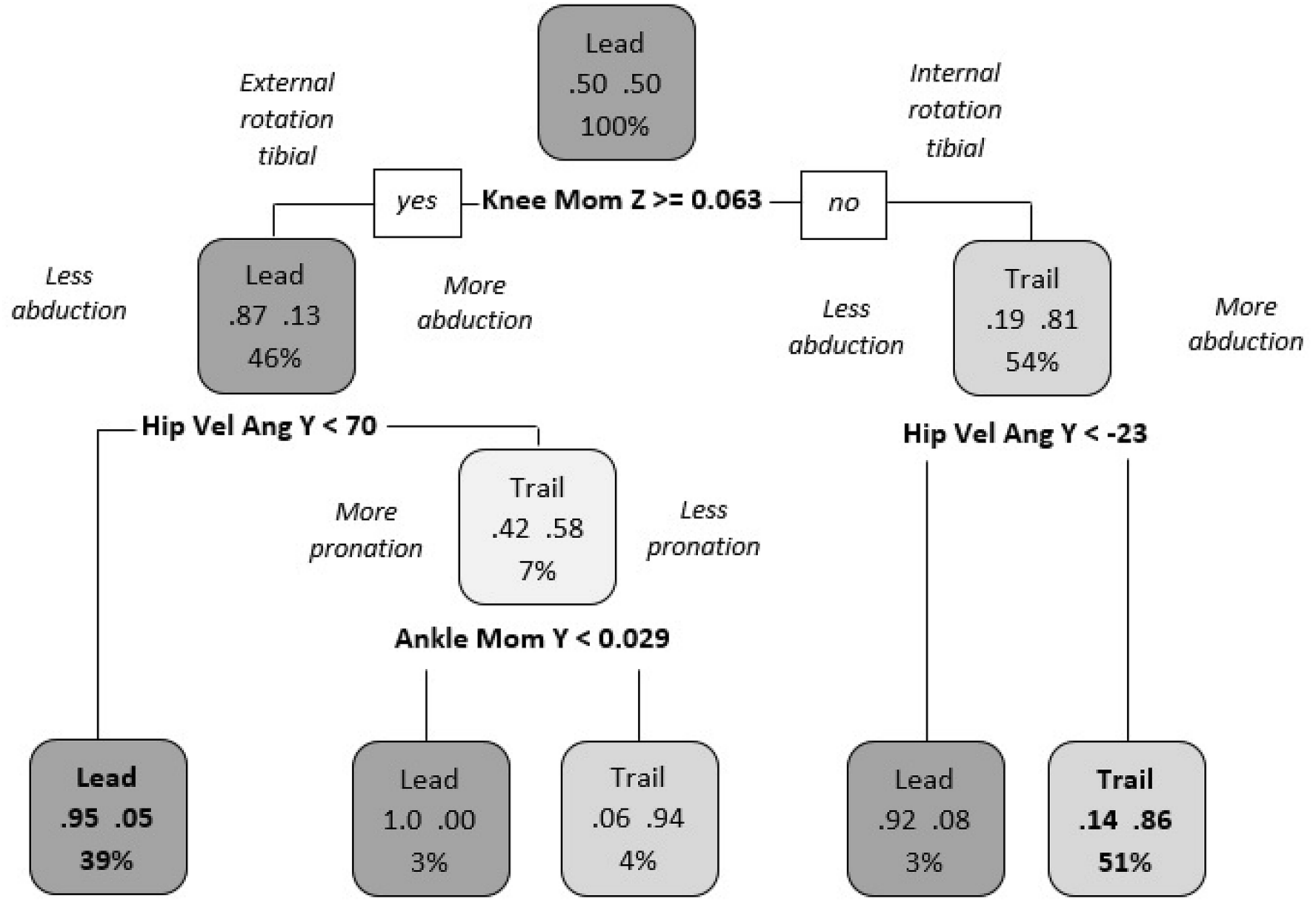
Differences between the lead and trail limbs in jump-landings when moving in the non-dominant direction. “Lead”: lead limb; “Trail”: trail limb; “Knee Mom Z”: Knee moment in the transverse plane; “Hip Vel Ang Y”: Hip angular velocity in the coronal plane; “Ankle Mom Y”: Ankle moment in the coronal plane.

Question 2 explored if significant differences exist between the lead and trail limbs in jump-landings when moving in the non-dominant direction. In this case we observed a prediction accuracy with both models (accuracy > 83.72%). Figure 4, shows that the lead limb strategy tends to less internal rotation of the tibia and lower hip abduction angular velocity in 39% of trials. Furthermore, in 51% of trials, the trail limb strategy tended towards a higher internal rotation of the tibia and greater hip abduction angular velocity. Questions 1 and 2 highlight that there were clear differences in the strategy between the lead and the trail limbs in a block jump-landing which were independent of the direction of movement.

**Figure 5:**
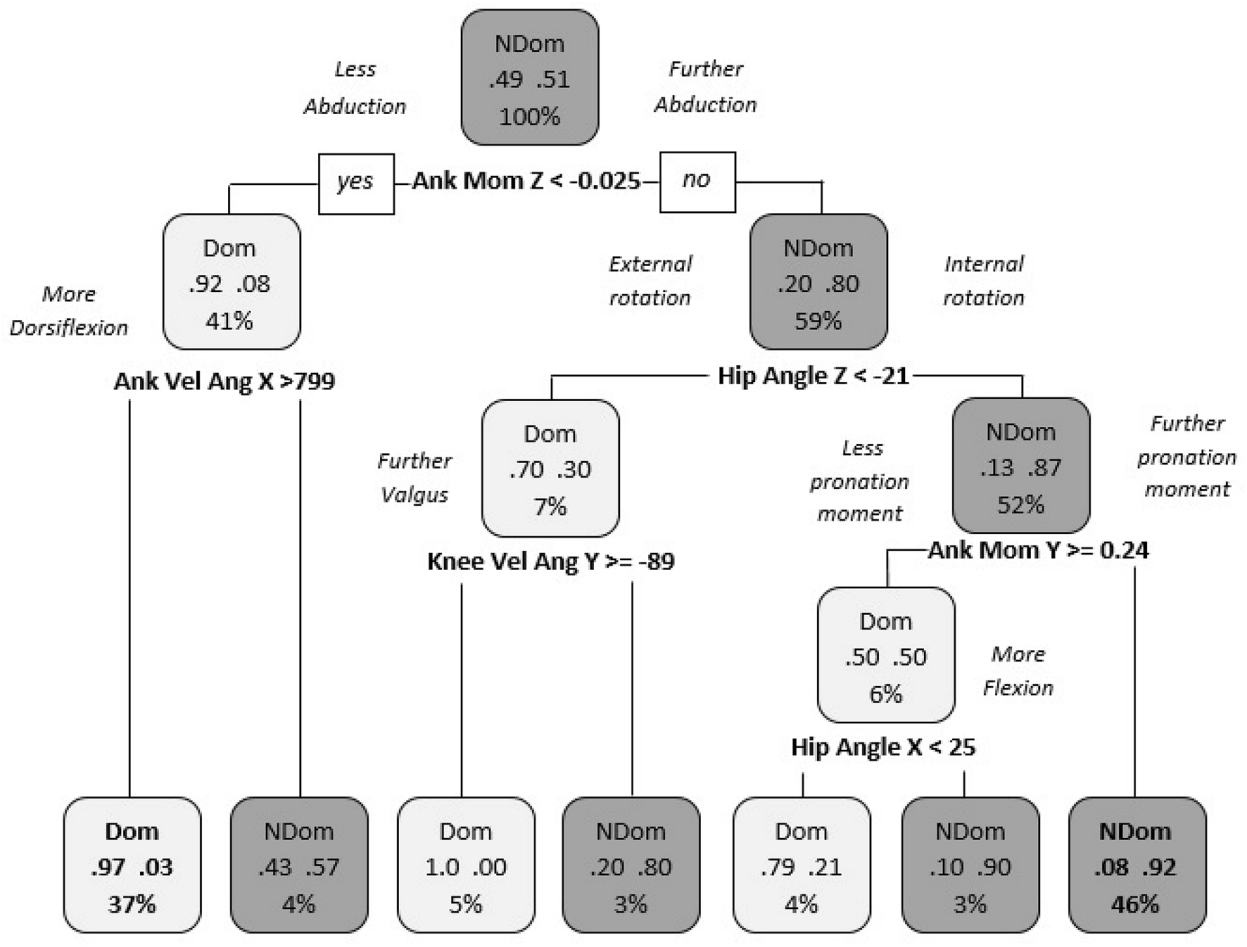
Differences between the dominant and non-dominant limb when both are performing the lead role. “NDom”: Non-dominant limb; “Dom”: Dominant limb; “Ank Mom Z”: Ankle moment in the transverse plane; “Ank Vel Ang X”: Ankle angular velocity in the sagittal plane; “Hip Angle Z”: Hip angle in the transverse plane; “Knee Vel Ang Y”: Knee angular velocity in the coronal plane; “Ank Mom Y”: Ankle moment in the coronal plane and “Hip Angle X”: Hip angle in the sagittal plane.

Question 3 considered if significant differences exist between dominant and non-dominant limbs when both are performing the lead role. Both models exhibited a predictive accuracy > 92.30% when comparing the lead limbs during jump-landing, indicating a difference in landing strategy between dominant and non-dominant limbs. Figure 5 showed that the dominant limb strategy tended towards a lower ankle abduction moment and a higher ankle dorsiflexion angular velocity in 37% of the trials. Moreover, in 46% of the trials, the non-dominant limb strategy tended towards a higher ankle abduction moment, a greater amount of hip internal rotation and a higher ankle pronation moment than the dominant limb.

**Figure 6:**
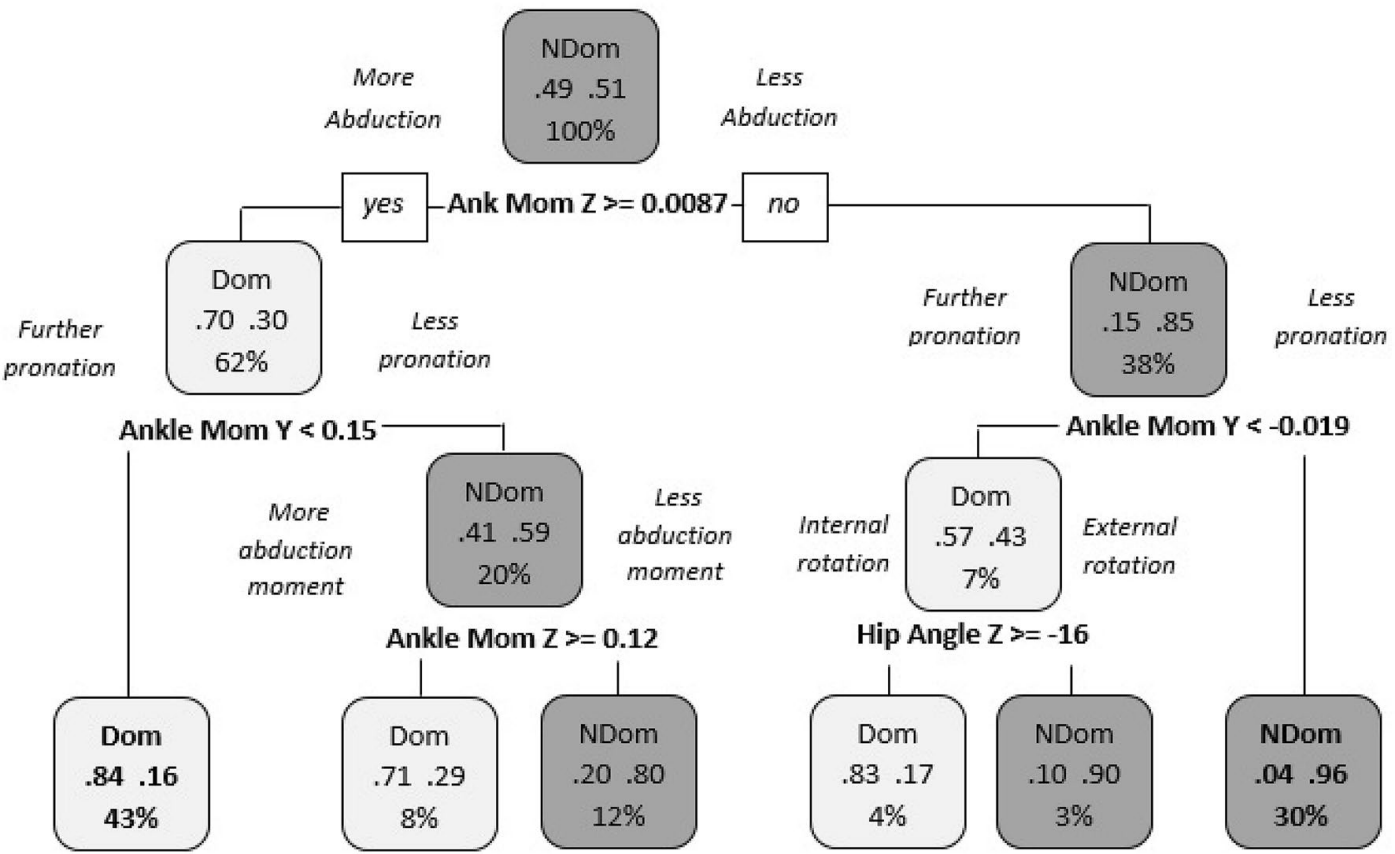
Differences between the dominant and non-dominant limb when both are performing the trail role. “NDom”: non-Dominant limb; “Dom”: Dominant limb; “Ank Mom Z”: Ankle moment in the transverse plane; “Ankle Mom Y”: Ankle moment in the coronal plane and “Hip Angle Z”: Hip angle in the transverse plane.

Finally, question 4 examined if significant differences exist between dominant and non-dominant limbs when both are performing the trail limb role. We observed a predictive accuracy of 91.21% indicating a difference in landing strategy. Figure 6 showed that the dominant limb strategy tended towards greater ankle abduction and pronation moments in 43% of the trials. Moreover, in 30% of the trials, the non-dominant limb strategy tended towards a lower ankle abduction and pronation moment than the dominant limb. Questions 3 and 4 demonstrate that the dominant and non-dominant limb had different strategies even when they are performing the same role independent of their position as the lead or the trail limb.

Due to space constrains only average results are shown below. We have setup a web page for the paper (https://dicits.ugr.es/papers/jlv) where the reader can find detailed experimental results.

## Discussion

Data for human movement are high-dimensional, heterogeneous and growing in volume, due to the access to improved technology. To harness the power of these data, and make research more effective and efficient, modern Machine Learning techniques complement traditional statistical tools. The interaction of Machine Learning and biomechanics, offers great promise, for advancing human movement research. As models become more complex, they also often become more difficult to interpret. In this manuscript, a detailed description of the movement strategies for the limbs was provided.

The results of this study suggest that there were differences in movement strategies when moving in the dominant and non-dominant directions for the dominant and non-dominant limbs when performing the lead or the trail limb. Moreover, Machine Learning offers an analysis technique capable of classifying the differences between limb movement strategies. This highlights the importance of considering limb dominance, limb role and direction of movement during block jump-landings, which could provide a greater understanding and identification of risky movements and preventative strategies for lower limb injuries and the improvement of performance during such tasks.

In volleyball, the dominant hand determines the three-step sequence technique to get the greatest spike, but when players perform a block jump-landing to the non-dominant direction, they change their natural three-step sequence and their limb movement strategies are modified. This seems to generate automatisms, which makes a condition of the block approach, and changes the movement strategy, depending on the direction of movement. Therefore, this may alter the muscle strength balance and promotes asymmetries^2^, and subsequently produces different movement strategies between limbs during jump-landing. Moreover, these asymmetries could be also accentuated due to an improper recovery from a previous injury or limb strength differences^3–5^. This gives us information about the differences of limbs movement strategies; therefore, coaches should train using strategies which minimize these asymmetries between limbs.

This current study created a protocol in which volleyball players performed a block jump-landing as fast as possible in a situation as ecologically valid as possible under laboratory conditions. Moreover, the majority of variables that have been previously reported as risk factors for lower limb injuries were integrated. In addition, it was considered some variables which have been less frequently included in sports performance analysis, including angular velocities and hip and ankle joint moments in the coronal and transverse planes. It was also considered that to achieve a greater understanding of movement strategies it is necessary to analyse the relationship between the joints in the different planes for the different limbs in a real game situation. Our results indicated that for all joints, regardless all joints, the multi-planar mechanism was crucial, when discerning between the dominant and the non-dominant limb strategies. Powers^29^ suggested that a combination of altered frontal and transverse plane motions of the hip would be expected to compound the loading of the iliotibial band. However, we found that the coronal and transverse planes have different roles in the different limbs which may have different effects on the underlying active and passive structures.

The results showed that there were different limb movement strategies, contrarily to previous studies which found symmetry between limbs^30,31^. In question 1, when comparing the lead and trail limbs, when the dominant limb performs as the trail limb and the non-dominant as the lead limb, we could see that the lead foot tends towards a greater ankle supination moment and hip abduction angle when compared to the trail limb. Moreover, in agreement with Hinshaw et al. (2018) we found that the lead limb had a higher VGRF than the trail limb, which could be related to the lead limb being the ipsolateral limb and consequently the limb which takes greater loads during landing, so these joints had to adapt to higher impact forces^18^. Contrarily, participants showed increased knee valgus moments for the trail limb when this role is performed by the dominant limb. In question 2, when comparing between the lead and trail limbs when the dominant limb is the lead limb and the non-dominant is the trail limb, it seems that the trail limb tends towards a higher tibial internal rotation than the lead limb. On the contrary, the lead limb tends towards a higher tibial external rotation and lower hip abduction angular velocity and less ankle supination than the trail limb. Therefore, it seems that the trail limb could have higher injury risk when moving to the non-dominant direction. An explanation for this could be that the trail limb corresponds with the non-dominant limb, which is the limb athletes tended to land on first when performing a spike^20^.

Moreover, in question 3, limb movement strategies were compared between the dominant and the non-dominant limb when they were performing their role as the lead limb. The non-dominant limb strategy tends towards a further ankle abduction and pronation moment and a higher hip internal rotation angle than the dominant limb. On the other hand, the dominant limb strategy tends towards a higher angular velocity for ankle dorsiflexion and knee valgus, and also a lower hip flexion angle. Thus, it seems that the key joints which coordinate the movement strategy when performs the lead limb for the non-dominant limb is the hip and the ankle, and for the dominant limb the knee and the ankle. In question 4, when comparing limb movement strategies between the dominant and the non-dominant limb when they were performing their role as the trail limb, the dominant limb tends towards a further ankle abduction and pronation moment than the non-dominant limb. Therefore, the identification of these variables could provide a greater understanding of specific preventative strategies for lower limb injuries and the improvement of performance during such tasks.

Performance analysis in sport science has experienced considerable recent changes, due largely to access to improved technology and increased applications from computer science^23^. We used Machine Learning methods to analyse all variables together during the phase of movement where injuries most frequently occur^8,32^. The ability to quantify differences between limbs and directions of movement using Machine Learning methods and the possibility to classify conditions with decision trees offers a valuable analysis. Future work may look to adopt, adapt and expand on current models associated with a specific sports movement to work towards flexible models for mainstream analysis and implementation^23^, and to establish the predictive performance of each specific technique/method^22^.

However, this study did have some limitations; only women from the same volleyball team were measured and lower limb movement were considered in the analysis, and finally, although participants moved as fast as possible, they had to control their jump-landings onto the force platforms, which does not replicate a real game situation. Future studies should consider other teams, levels and men. The analysis of which variables show the greatest influence in the different models may offer a better understanding of how the individual joints of the lower limbs act during a block jump-landing and how these may be associated with potential joint overload which could produce injury risk and could help to improve performance.

## Conclusions

It is necessary to consider limb role, directions of movement and limb dominance due to the differences seen between limb movement strategies during block jump-landings. These require protocols to be as ecologically valid as possible in order to explore such differences. Moreover, the use of Machine Learning methods may in turn have practical applications for coaches and trainers and allow a greater awareness of the individual movements between limbs and directions of movement. Machine Learning models can help build effective non-linear relationships between data can be learned which could analyse more complex patterns. All these detailed and valuable descriptions of kinematic and kinetic variables which were given could provide relevant information about how to improve the performance of the players and how to plan the training in order to avoid an overload that could lead to risk of injury. This highlights that, there is a necessity to consider the learning models, in which the spike approach unilaterally is taught before the block approach (bilaterally). Therefore, we support the idea of teaching bilateral approach before learning the spike, in order to improve coordination and to avoid asymmetries between limbs.

## Data Availability

The data sets used and/or analysed during this study are available from the corresponding author on reasonable request.
